# Efficacy of continued cetuximab for unresectable metastatic colorectal cancer after disease progression during first-line cetuximab-based chemotherapy: a retrospective cohort study

**DOI:** 10.18632/oncotarget.7193

**Published:** 2016-02-04

**Authors:** Qingyang Feng, Ye Wei, Li Ren, Peng Zheng, Yiyi Yu, Qinghai Ye, Jianyong Ding, Jingwen Chen, Wenju Chang, Yunshi Zhong, Dexiang Zhu, Qi Lin, Liangliang Yang, Xinyu Qin, Jianmin Xu

**Affiliations:** ^1^ Department of General Surgery, Zhongshan Hospital, Fudan University, Shanghai, China; ^2^ Department of Medical Oncology, Zhongshan Hospital, Fudan University, Shanghai, China; ^3^ Department of Liver Surgery, Zhongshan Hospital, Fudan University, Shanghai, China; ^4^ Department of Thoracic Surgery, Zhongshan Hospital, Fudan University, Shanghai, China

**Keywords:** colorectal cancer, metastasis, cetuximab, cross-line treatment, early tumor shrinkage

## Abstract

This study assessed second-line continued use of cetuximab for treatment of unresectable metastatic colorectal cancer (mCRC) after disease progression during first-line cetuximab-based therapy. Consecutive patients with wild-type KRAS exon 2 and unresectable mCRC were retrospectively enrolled after disease progression during first-line cetuximab-based chemotherapy. Second-line continued cetuximab plus changed chemotherapy (cetuximab continuation group, *n* = 102) was compared with changed chemotherapy only (chemotherapy only group, *n* = 96) with respect to treatment efficacy and safety endpoints. NRAS and other KRAS genotypes were also detected as a post hoc analysis. The cetuximab continuation group showed better progression-free survival (median, 6.3 vs. 4.5 months, *P* = 0.004), overall survival (median, 17.3 vs. 14.0 months, *P* < 0.001) and disease control rate (70.6% vs. 53.1%, *P* = 0.011), and a potentially better overall response rate (18.6% vs. 9.4%, *P* = 0.062) than the chemotherapy only group. These benefits were seen mainly in patients with all RAS wild-type and exhibiting first-line early tumor shrinkage (ETS). For patients with other RAS mutations or who did not achieve first-line ETS, there was no difference between the two groups. These findings suggest that for patients with all RAS wild-type and unresectable mCRC who had disease progression during first-line cetuximab-based treatment, second-line continued cetuximab is effective. Moreover, ETS during first-line cetuximab-based treatment may be predictive of the efficacy of second-line continued cetuximab.

## INTRODUCTION

Metastatic colorectal cancer (mCRC) is a major healthcare problem globally [1]. During the course of their disease, approximately half of patients with colorectal cancer will develop distant metastasis [2], which is the major cause of death. If feasible, radical resection is the ideal treatment for mCRC, but in the majority of patients, mCRC is unresectable, even after intensive treatment with targeted agents plus chemotherapy [3].

Cetuximab is a promising agent that targets epidermal growth factor receptor (EGFR) and has shown an impressive ability to improve the tumor response and increase progression-free survival (PFS) and overall survival (OS) among patients with all RAS wild-type mCRC [4–6]. It also significantly increases the number of patients with inoperable metastases whose tumors become resectable after treatment [3]. However, for patients who fail to respond to standard first-line cetuximab-based chemotherapy, it is unclear whether continuation of cetuximab would provide any benefit as part of second-line combination therapy. Traditionally, anti-EGFR antibodies, including cetuximab, have not been considered suitable for continued use after disease progression. But the clinical evidence supporting that presumption is not compelling.

In this study, consecutive patients with wild-type KRAS exon 2 (codon 12/13) and unresectable mCRC were retrospectively enrolled after disease progression during first-line treatment with cetuximab plus chemotherapy. In the second-line treatment, the efficacy and safety were compared between continued cetuximab plus changed chemotherapy and changed chemotherapy only. NRAS and other KRAS (referred to as other RAS) genotypes were detected as a post hoc analysis. Subgroup analysis was also conducted to find patients most likely to benefit from cross-line treatment with cetuximab.

## RESULTS

### Patient characteristics

**Figure 1:**
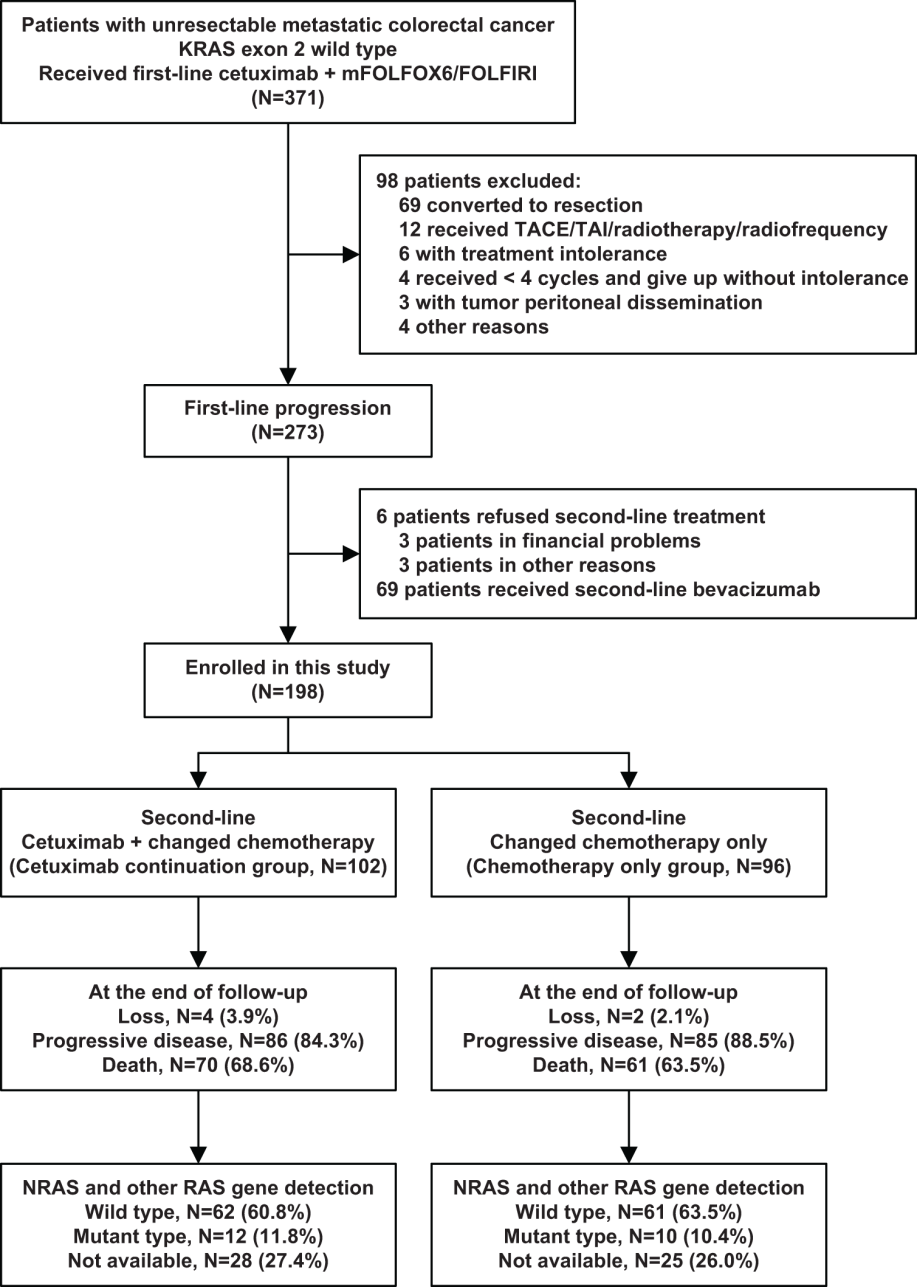
Patients enrollment mFOLFOX6: fluorouracil, leucovorin, and oxaliplatin; FOLFIRI: fluorouracil, leucovorin, and irinotecan; TACE: transcatheter arterial chemoembolization; TAI: transcatheter arterial infusion.

A total of 198 eligible patients who exhibited disease progression during first-line cetuximab-based chemotherapy were ultimately included in the study: 102 patients in the cetuximab continuation group (receiving second-line continued cetuximab plus changed chemotherapy) and 96 patients in the chemotherapy only group (only receiving second-line changed chemotherapy). The tumor samples were re-collected to detect other RAS genotypes (Figure 1). The demographic, clinical characteristics and other RAS genotypes were balanced between the two groups, as shown in Table 1. The median follow-up time of all patients was 12.9 months (IQR = [10.0–17.3]) from the start of second-line treatment, and was 24.9 months (IQR = [19.6–30.7]) from the start of first-line treatment. Six (3.0%) patients were lost to follow-up by the end: 4 (3.9%) patients in cetuximab continuation group and 2 (2.1%) patients in chemotherapy only group, which was not a significant difference between groups.

**Table 1:** Baseline characteristics of the patients

	Cetuximab continuation (*N* = 102)	Chemotherapy only (*N* = 96)	*P* value
Age - year			
Mean ± SD	54.6 ± 7.4	54.6 ± 10.7	0.966
Range - no. (%)			0.993
≤ 55	52 (51.0)	49 (51.0)	
> 55	50 (49.0)	47 (49.0)	
Sex - no. (%)			0.549
Male	71 (69.6)	63 (65.6)	
Female	31 (30.4)	33 (34.4)	
ECOG performance status - no. (%)			0.632
0	39 (38.2)	41 (42.7)	
1	52 (51.0)	48 (50.0)	
2	11 (10.8)	7 (7.3)	
Primary tumor site - no. (%)			0.717
Right-sided colon	33 (32.4)	26 (27.1)	
Left-sided colon	30 (29.4)	31 (32.3)	
Rectum	39 (38.2)	39 (40.6)	
No. of organs with metastases - no. (%)			0.781
1	78 (76.5)	75 (78.1)	
≥ 2	24 (23.5)	21 (21.9)	
Organs with metastases - no. (%)			0.888
Liver only	73 (71.6)	70 (72.9)	
Liver plus others	22 (21.6)	21 (21.9)	
Non-liver	7 (6.9)	5 (5.2)	
No. of liver metastatic lesions - no. (%)			0.829
1–2	7 (7.4)	9 (9.9)	
3–5	43 (45.3)	40 (44.0)	
> 5	45 (47.4)	42 (46.1)	
Other RAS genotype - no. (%)			0.915
Wild-type	62 (60.8)	61 (63.5)	
Mutant type	12 (11.8)	10 (10.4)	
Not available	28 (27.4)	25 (26.0)	
BRAF mutation - no. (%)	8 (7.8)	7 (7.3)	0.883
Primary tumor resection - no. (%)	73 (71.6)	72 (75.0)	0.586

SD: standard deviation; ECOG: Eastern Cooperative Oncology Group.

Other RAS including KRAS exons 3 (codon 59 and 61) and exon 4 (codon 117 and 146), NRAS exons 2 (codon 12 and 13), exon 3 (codon 59 and 61) and 4 (codon 117 and 146).

### Treatment exposure

Before enrollment, the first-line treatment was balanced between the two groups, with no significant differences in PFS (*P* = 0.796), early tumor shrinkage (ETS) rate (*P* = 0.821), ORR (*P* = 0.951) or receiving maintenance treatment (*P* = 0.661) during the period of first-line treatment. For second-line treatment, the chemotherapy regimen was changed for all patients. After second-line disease progression, no significant difference was observed between the two groups whether they received TACE/TAI, radiofrequency ablation, radiotherapy or chemotherapy. However, there tended be more patients in the cetuximab continuation group receiving bevacizumab following treatment (*P* = 0.052). Details are shown in Table 2.

**Table 2:** Treatment exposure

	Cetuximab continuation (*N* = 102)	Chemotherapy only (*N* = 96)	*P* value
In first-line treatment			
Chemotherapy regimen - no. (%)			0.846
mFOLFOX6	63 (61.8)	58 (60.4)	
FOLFIRI	39 (38.2)	38 (39.6)	
Maintenance treatment - no. (%)	30 (29.4)	31 (32.2)	0.661
PFS - month (log-rank test)			0.796
Median	11.0	11.2	
95% CI	9.6–12.4	10.2–12.2	
Achieved early tumor shrinkage - no. (%)	43 (42.2)	42 (43.8)	0.821
Overall response - no. (%)			0.718
CR	3 (2.9)	5 (5.2)	
PR	55 (53.9)	50 (52.1)	
SD or PD	44 (43.1)	41 (42.7)	
Overall response rate - %	56.9	57.3	0.951
Acne-like rash, grade - no. (%)			0.681
0–1	63 (61.8)	62 (64.6)	
≥ 2	39 (38.2)	34 (35.4)	
In second-line treatment			
Chemotherapy regimen - no. (%)			0.846
mFOLFOX6	39 (38.2)	38 (39.6)	
FOLFIRI	63 (61.8)	58 (60.4)	
Maintenance treatment - no. (%)	15 (14.7)	8 (8.3)	0.162
After second-line progression			
TACE/TAI for metastases - no. (%)	27 (26.5)	26 (27.1)	0.922
Radiofrequency for metastases - no. (%)	10 (9.8)	13 (13.5)	0.412
Radiotherapy for metastases - no. (%)	9 (8.8)	5 (5.2)	0.321
Following chemotherapy - no. (%)			0.273
Not PD^#^	16 (15.7)	11 (11.5)	
Intensive chemotherapy^§^	59 (57.8)	48 (50.0)	
Best support care	19 (18.6)	23 (24.0)	
Not known	8 (7.8)	14 (14.6)	
Following bevacizumab - no. (%)	18 (17.6)	8 (8.3)	**0.052**

CI: confidence interval; Early tumor shrinkage: 8 weeks, shrinkage > / = 20% of the tumor; CR: complete response; PR: partial response; SD: stable disease; PD: progressive disease; TACE: transcatheter arterial chemoembolization; TAI: transcatheter arterial infusion.

# Not PD: not reaching progressive disease by the end of follow-up.

§ Intensive chemotherapy: receiving at least one intravenous chemotherapy medicine.

Bold font for *P* < 0.10.

### Efficacy of second-line treatment

**Figure 2:**
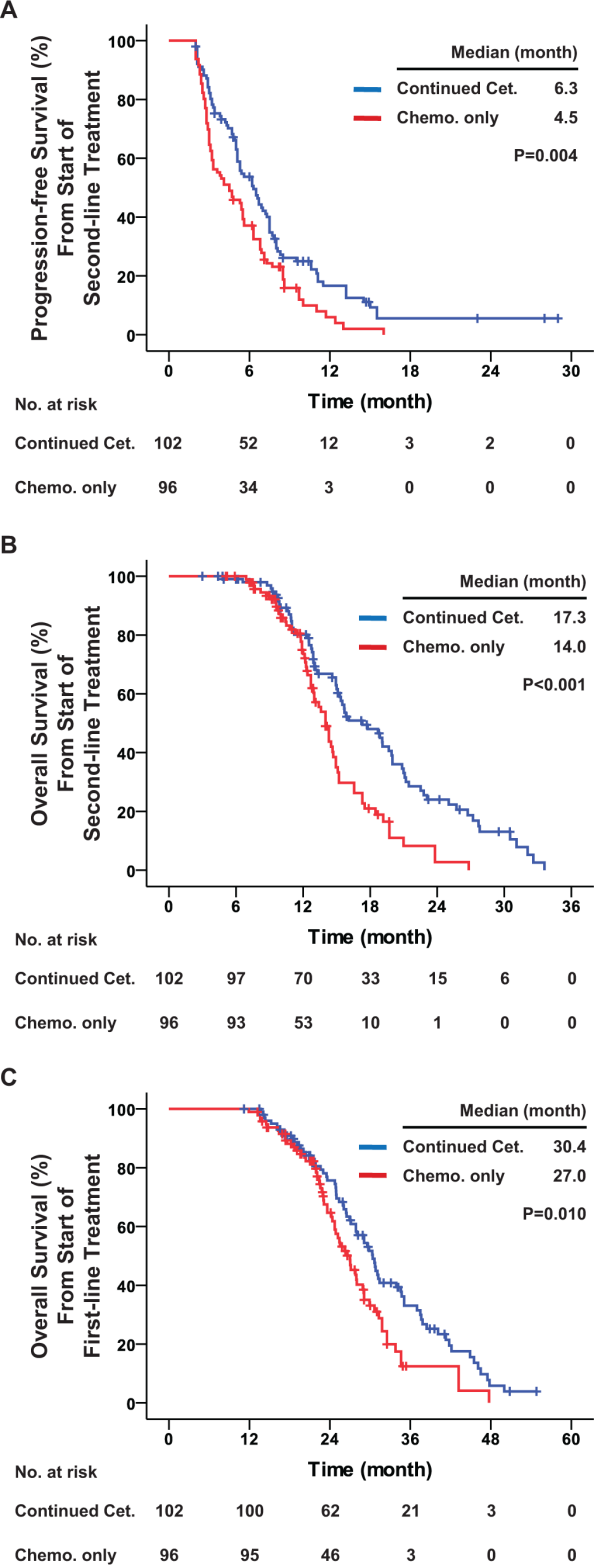
Progression-free survival and overall survival among all patients with KRAS exon 2 wild-type Kaplan-Meier curves for (**A**) progression-free survival from the start of second-line treatment, (**B**) overall survival from start of second-line treatment, and (**C**) overall survival from start of first-line treatment. The curves compare continued cetuximab plus changed chemotherapy and changed chemotherapy only as second-line treatment in all patients. Cet.: Cetuximab; Chemo.: chemotherapy. *P* values were determined using the log-rank test.

All 198 patients included in this study had KRAS exon 2 wild-type. Among them 171 (86.4%) experienced disease progression during second-line treatment: 86 (84.3%) in the cetuximab continuation group and 85 (88.5%) in the chemotherapy only group. The median PFS from the start of second-line treatment was 6.3 months in the cetuximab continuation group, which was significantly better than the 4.5 months in the chemotherapy only group (hazard ratio = 0.646, *P* = 0.004). In terms of OS, a total of 131 (66.2%) deaths occurred by the end of follow-up: 70 (68.6%) in the cetuximab continuation group and 61 (63.5%) in the chemotherapy only group. All of these patients died of mCRC. The median OS from the start of second-line treatment was 17.3 months in the cetuximab continuation group, which was significantly better than the 14.0 months in the chemotherapy only group (hazard ratio = 0.503, *P* < 0.001). In addition, the cetuximab continuation group had significantly better OS from the start of first-line treatment than the chemotherapy only group (median, 30.4 vs. 27.0 months, hazard ratio = 0.629, *P* = 0.010). In terms of tumor response, the cetuximab continuation group had significantly better DCR (70.6% vs. 53.1%, odds ratio = 2.118, *P* = 0.011) and potentially better ORR (18.6% vs. 9.4%, odds ratio = 2.213, *P* = 0.062) than the chemotherapy only group (Table 3 and Figure 2).

**Table 3:** Efficacy of second-line treatment among all patients with KRAS exon 2 wild-type

	Total patients		First-line ETS		First-line NOT ETS	
	Cetuximab continuation (*N* = 102)	Chemo. only (*N* = 96)	Cetuximab continuation (*N* = 43)	Chemo. only (*N* = 42)	Cetuximab continuation (*N* = 59)	Chemo. only (*N* = 54)
PFS - month						
Median	6.3	4.5	7.7	4.5	5.0	4.1
95% CI	5.1–7.5	2.9–6.1	7.0–8.4	2.0–7.0	4.4–5.6	2.3–5.9
Hazard ratio	0.646		0.377		0.925	
95% CI	0.476–0.877		0.223–0.637		0.623–1.374	
*P* value (log-rank test)	0.004*		< 0.001*		0.695	
OS - month						
Median	17.3	14.0	21.1	14.3	15.3	13.4
95% CI	14.2–20.4	13.1–14.9	18.4–23.8	13.0–15.6	14.1–16.4	11.8–15.0
Hazard ratio	0.503		0.258		0.740	
95% CI	0.348–0.727		0.123–0.544		0.480–1.140	
*P* value (log-rank test)	< 0.001*		< 0.001*		0.168	
Overall response - no. (%)						
CR	1 (1.0)	0 (0)	1 (2.3)	0 (0)	0 (0)	0 (0)
PR	18 (17.6)	9 (9.4)	14 (32.6)	7 (16.7)	4 (6.8)	2 (3.7)
SD	53 (52.0)	42 (43.8)	22 (51.2)	16 (38.1)	31 (52.5)	26 (48.1)
PD	27 (26.5)	44 (45.8)	4 (9.3)	19 (45.2)	23 (39.0)	25 (46.3)
Not evaluable	3 (2.9)	1 (1.0)	2 (4.7)	0 (0)	1 (1.7)	1 (1.9)
Overall response rate - (%)	18.6	9.4	34.9	16.7	6.8	3.7
Odds ratio	2.213		2.679		1.891	
95% CI	0.948–5.168		0.960–7.470		0.332–10.765	
*P* value	**0.062**		**0.055**		0.681	
Disease control rate - (%)	70.6	53.1	86.0	54.8	59.3	51.9
Odds ratio	2.118		5.094		1.354	
95% CI	1.180–3.801		1.774–14.632		0.643–2.852	
*P* value	0.011*		0.002*		0.425	

Chemo.: chemotherapy; ETS: early tumor shrinkage (8 weeks, shrinkage ≥ 20% of the tumor); PFS: progression-free survival; OS: overall survival; CI: confidence interval; CR: complete response; PR: partial response; SD: stable disease; PD: progressive disease.

Overall response rate = CR + PR; Disease control rate = CR + PR + SD.

*P* values of “Overall response rate” and “Disease control rate” were calculated using two-sided Pearson's χ^2^ tests or Fisher's exact test for any cell expected count less than 5 or sample size less than 40.

Bold font for *P* < 0.10;

* for *P* < 0.05.

**Figure 3:**
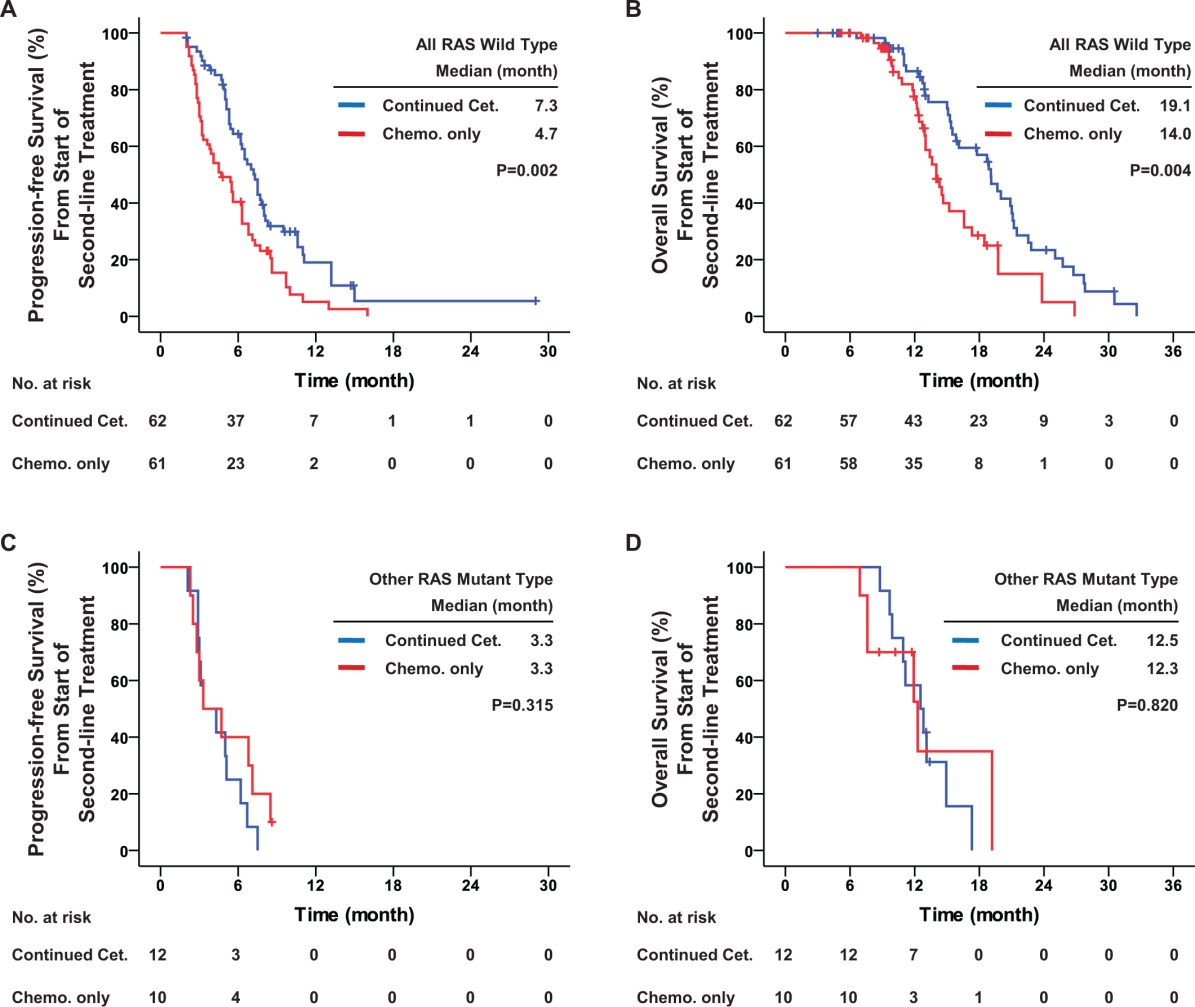
Progression-free survival and overall survival among patients receiving other RAS detection Kaplan-Meier curves for (**A**) progression-free survival and (**B**) overall survival among patients with all RAS wild-type; and for (**C**) progression-free survival and (**D**) overall survival among patients with other RAS mutant type. The curves compare continued cetuximab plus changed chemotherapy and changed chemotherapy only as second-line treatment. Cet.: Cetuximab; Chemo.: chemotherapy. *P* values were determined using the log-rank test.

From all patients, 145 (73.2%) samples were available for other RAS gene detection: 123 (62.1%) with all RAS wild-type and 22 (11.1%) with other RAS mutations. During the first-line cetuximab-based treatment, patients with all RAS wild-type had significantly better PFS than patients with other RAS mutations (median, 11.4 vs. 7.5 months, hazard ratio = 0.575, *P* = 0.015, as shown in Supplementary Figure S1). After first-line disease progression, other RAS mutations were still a strong contraindication for continuation of cetuximab, with a significant interaction (*P* = 0.029). For patients with all RAS wild-type disease, second-line continued cetuximab significantly improved second-line PFS (median, 7.3 vs. 4.7 months, hazard ratio = 0.538, *P* = 0.002), OS (median, 19.1 vs. 14.0 months, hazard ratio = 0.502, *P* = 0.004), DCR (83.9% vs. 57.4%, odds ratio = 3.863, *P* = 0.001) and ORR (21.0% vs. 8.2%, odds ratio = 2.971, *P* = 0.045) as compared with second-line chemotherapy only. However, for patients with other RAS mutations, no significant benefit in PFS, OS, ORR or DCR was obtained from the second-line continued cetuximab (Table 4 and Figure 3).

**Table 4:** Efficacy of second-line treatment among patients receiving other RAS detection

	Patients with all RAS wild-type						Patients with other RAS mutant type	
	All		First-line ETS		First-line NOT ETS			
	Cetuximab continuation (*N* = 62)	Chemo. only (*N* = 61)	Cetuximab continuation (*N* = 28)	Chemo. only (*N* = 28)	Cetuximab continuation (*N* = 34)	Chemo. only (*N* = 33)	Cetuximab continuation (*N* = 12)	Chemo. only (*N* = 10)
PFS - month								
Median	7.3	4.7	8.0	5.4	5.6	4.5	3.3	3.3
95% CI	6.3–8.3	3.2–6.2	7.2–8.8	3.6–7.2	4.4–6.8	2.9–6.1	1.3–5.3	0.7–5.9
Hazard ratio	0.538		0.315		0.739		1.590	
95% CI	0.362–0.800		0.164–0.604		0.432–1.265		0.632–4.001	
*P* value (log-rank test)	0.002*		*<* 0.001*		0.264		0.315	
OS - month								
Median	19.1	14.0	21.0	14.3	16.1	13.4	12.5	12.3
95% CI	16.9–21.3	12.8–15.2	19.6–22.4	12.4–16.2	13.3–18.9	12.6–14.2	9.7–15.4	7.3–17.2
Hazard ratio	0.502		0.239		0.725		1.134	
95% CI	0.311–0.810		0.092–0.623		0.404–1.302		0.383–3.355	
*P* value (log-rank test)	0.004*		0.002*		0.277		0.820	
Overall response - no. (%)								
CR	1 (1.6)	0 (0)	1 (3.6)	0 (0)	0 (0)	0 (0)	0 (0)	0 (0)
PR	12 (19.4)	5 (8.2)	9 (32.1)	4 (14.3)	3 (8.8)	1 (3.0)	0 (0)	1 (10.0)
SD	39 (62.9)	30 (49.2)	17 (60.7)	13 (46.4)	22 (64.7)	17 (51.5)	6 (50.0)	4 (40.0)
PD	8 (12.9)	25 (41.0)	0 (0)	11 (39.3)	8 (23.5)	14 (42.4)	6 (50.0)	5 (50.0)
Not evaluable	2 (3.2)	1 (1.6)	1 (3.6)	0 (0)	1 (2.9)	1 (3.0)	0 (0)	0 (0)
Overall response rate - (%)	21.0	8.2	35.7	14.3	8.8	3.0	0	10.0
Odds ratio	2.971		3.333		3.097		NE	
95% CI	0.989–8.930		0.899–12.363		0.305–31.400		NE	
*P* value	0.045*		**0.064**		0.614		0.455	
Disease control rate - (%)	83.9	57.4	96.4	60.7	73.5	54.5	50.0	50.0
Odds ratio	3.863		17.471		2.315		1.000	
95% CI	1.658–9.001		2.065–147.774		0.831–6.450		0.187–5.357	
*P* value	0.001*		0.001*		0.105		1.000	

Chemo.: chemotherapy; ETS: early tumor shrinkage (8 weeks, shrinkage ≥ 20% of the tumor); PFS: progression-free survival; OS: overall survival; CI: confidence interval; CR: complete response; PR: partial response; SD: stable disease; PD: progressive disease; NE: not evaluable.

Overall response rate = CR + PR; Disease control rate = CR + PR + SD.

Other RAS including KRAS exons 3 (codon 59 and 61) and exon 4 (codon 117 and 146), NRAS exons 2 (codon 12 and 13), exon 3 (codon 59 and 61) and 4 (codon 117 and 146).

*P* values of “Overall response rate” and “Disease control rate” were calculated using two-sided Pearson's χ^2^ tests or Fisher's exact test for any cell expected count less than 5 or sample size less than 40.

Bold font for *P <* 0.10;

* for *P <* 0.05.

As a retrospective study without randomization, some potential imbalances between the two groups could interfere with analysis of the efficacy of second-line continued cetuximab. Therefore, multivariate Cox regression analysis was conducted for confirmation. The results showed that first-line ETS (hazard ratio = 0.682, *P* = 0.041) and second-line continued cetuximab (hazard ratio = 0.675, *P* = 0.016) were independent protective factors for second-line PFS; other RAS mutant type (hazard ratio = 2.141, *P* = 0.003) and BRAF mutant type (hazard ratio = 3.001, *P* < 0.001) were independent risk factors. No significance was detected for primary tumor site, organs with metastases and first-line chemotherapy regimen, among others (Supplementary Table S1).

### Subgroup analysis for second-line treatment

**Figure 4:**
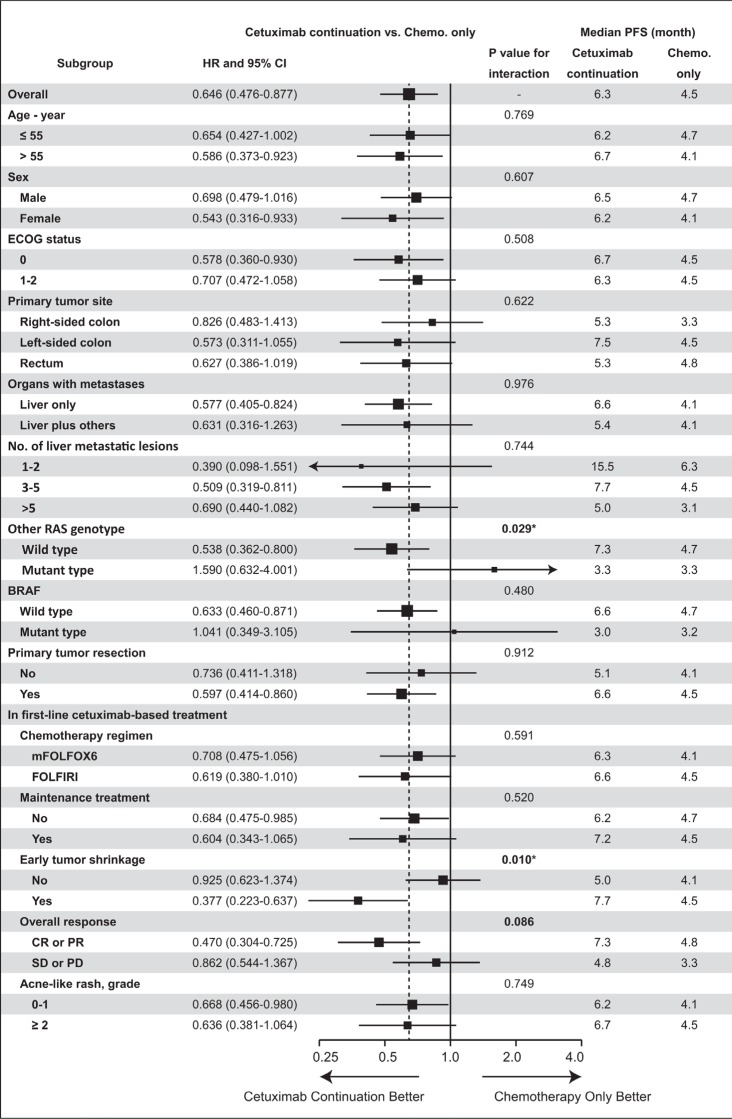
Subgroup analysis of second-line progression-free survival PFS: progression-free survival; CI: confidence interval; ECOG: Eastern Cooperative Oncology Group; Early Tumor Shrinkage: 8 weeks, shrinkage ≥ 20% of the tumor; CR: complete response; PR: partial response; SD: stable disease; PD: progressive disease; *P* value: interaction analysis with Cox regression. Bold font for *P* < 0.10; * for *P* < 0.05.

Subgroup analysis was conducted to find more accurate predictors for second-line continued use of cetuximab after progression during first-line cetuximab-based treatment. All potential factors were included in the planned subgroup analysis of PFS from the start of second-line treatment (as shown in Figure 4). ETS in first-line cetuximab-based treatment was demonstrated to be predictive of the efficacy of second-line continued cetuximab, with significant interaction (*P* = 0.010).

**Figure 5:**
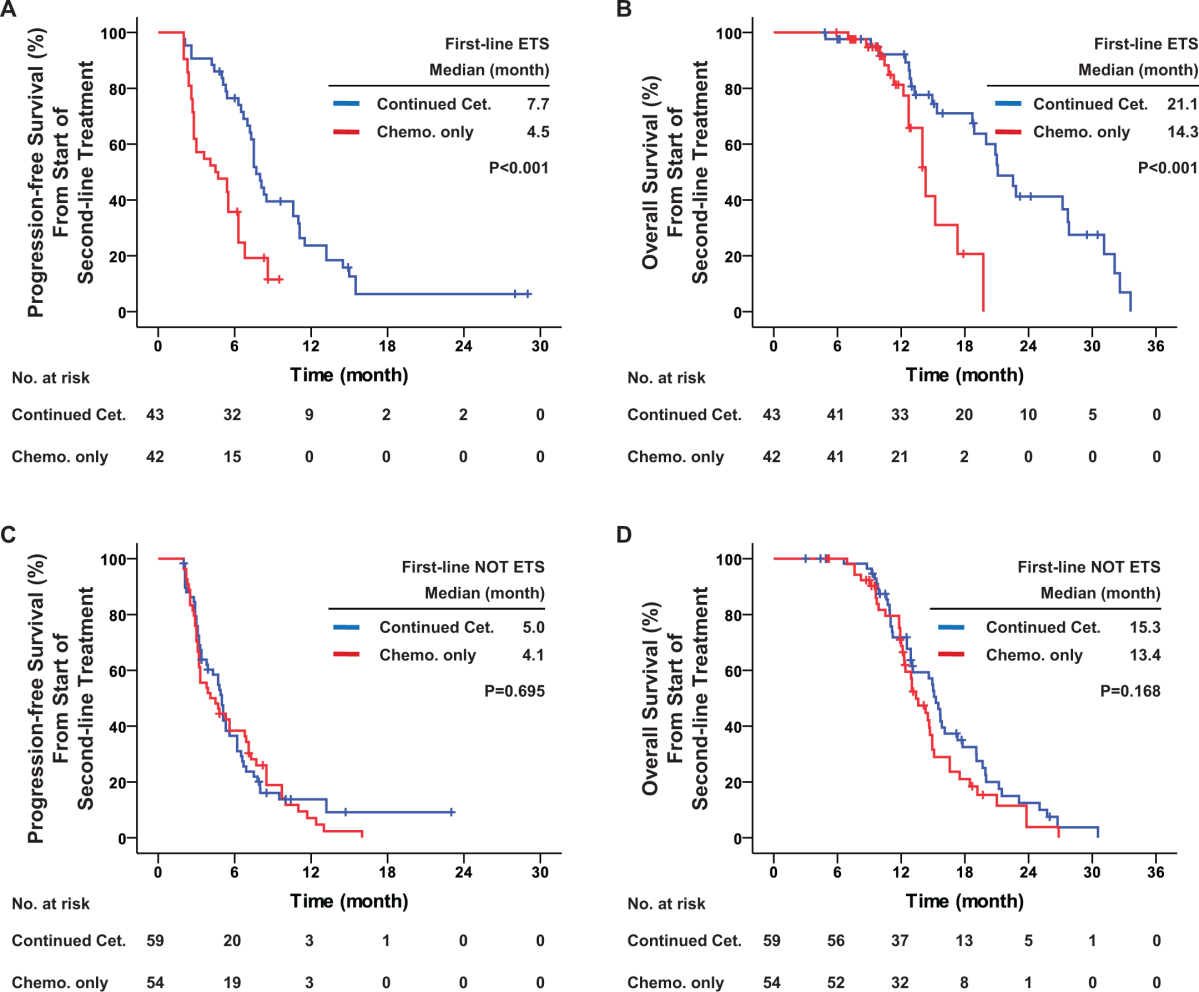
First-line early tumor shrinkage was predictive of the efficacy of second-line treatment in all patients with KRAS exon 2 wild-type Kaplan-Meier curves for (**A**) progression-free survival and (**B**) overall survival among patients with KRAS exon 2 wild-type and who achieved early tumor shrinkage during first-line treatment; and for (**C**) progression-free survival and (**D**) overall survival among patients with KRAS exon 2 wild-type but who did not achieve early tumor shrinkage during first-line treatment. The curves compare continued cetuximab plus changed chemotherapy and changed chemotherapy only as second-line treatments. Cet.: Cetuximab; Chemo.: chemotherapy; ETS: early tumor shrinkage. *P* values were determined using the log-rank test.

Among all patients with KRAS exon 2 wild-type included in this study, 85 (42.9%) achieved ETS during first-line treatment. From start of second-line treatment in these patients, continued cetuximab significantly improved PFS (median, 7.7 vs. 4.5 months, hazard ratio = 0.377, *P* < 0.001), OS (median, 21.1 vs. 14.3 months, hazard ratio = 0.258, *P* < 0.001) and DCR (86.0% vs. 54.8%, odds ratio = 5.094, *P* = 0.002), and potentially improved ORR (34.9% vs. 16.7%, odds ratio = 2.679, *P* = 0.055) as compared with chemotherapy alone. In patients who did not reach ETS during first-line treatment, from start of second-line treatment no significant difference was observed between the two groups with respect to PFS (median, 5.0 vs. 4.1 months, hazard ratio = 0.925, *P* = 0.695), OS (median, 15.3 vs. 13.4 months, hazard ratio = 0.740, *P* = 0.168), DCR (59.3% vs. 51.9%, odds ratio = 1.354, *P* = 0.425) and ORR (6.8% vs. 3.7%, odds ratio = 1.891, *P* = 0.681) (Table 3 and Figure 5).

**Figure 6:**
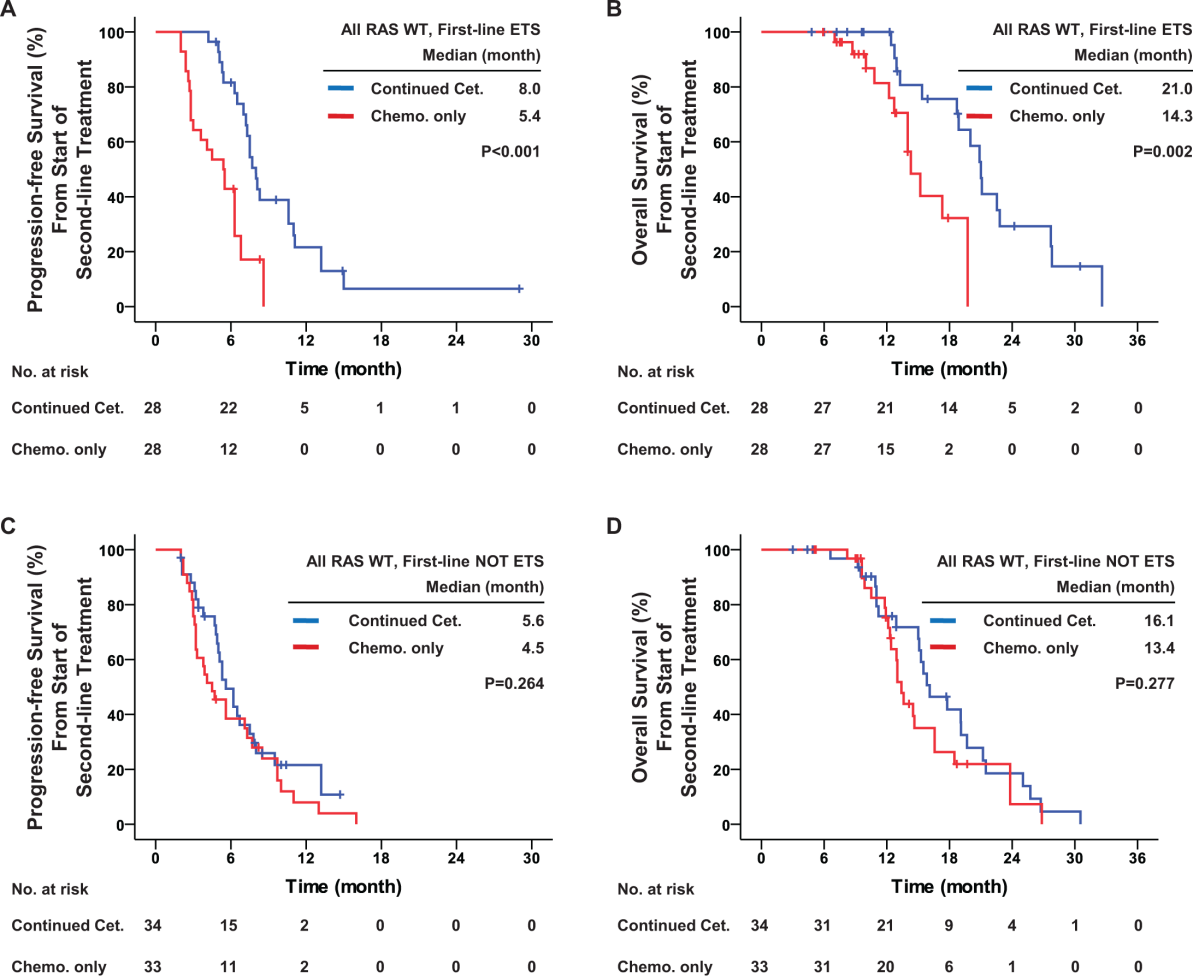
First-line early tumor shrinkage was predictive of the efficacy of second-line treatment in patients with all RAS wild-type Kaplan-Meier curves for (**A**) progression-free survival and (**B**) overall survival among patients with all RAS wild-type and who achieved early tumor shrinkage during first-line treatment; and for (**C**) progression-free survival and (**D**) overall survival among patients with all RAS wild-type but who did not achieve early tumor shrinkage during first-line treatment. The curves compare the continued cetuximab plus changed chemotherapy and changed chemotherapy only as second-line treatment. Cet.: Cetuximab; Chemo.: chemotherapy; ETS: early tumor shrinkage. *P* values were determined using the log-rank test.

For the patients with all RAS wild-type, first-line ETS was still predictive of the efficacy of second-line continued cetuximab, with a potentially significant interaction (*P* = 0.076). For patients with all RAS wild-type and first-line ETS, second-line continued cetuximab significantly improved PFS (median, 8.0 vs. 5.4 months, hazard ratio = 0.315, *P* < 0.001), OS (median, 21.0 vs. 14.3, hazard ratio = 0.239, *P* = 0.002) and DCR (96.4% vs. 60.7%, odds ratio = 17.471, *P* = 0.001), and potentially improved ORR (35.7% vs. 14.3%, odds ratio = 3.333, *P* = 0.064) as compared with chemotherapy alone. For patients with all RAS wild-type but without first-line ETS, no significant difference was observed between the two groups (Table 4 and Figure 6).

The first-line overall response was also a potential predictor of the efficacy of second-line continued cetuximab, with interaction of PFS from the start of second-line treatment (*P* = 0.086). For patients who reached first-line CR or PR, continued cetuximab significantly improved second-line PFS (median, 7.3 vs. 4.8 months, hazard ratio = 0.470, *P* < 0.001), OS (median, 20.0 vs. 14.3 months, hazard ratio = 0.408, *P* = 0.001) and DCR (81.0% vs. 60.0%, odds ratio = 2.848, *P* = 0.014), and potentially improved ORR (25.9% vs. 12.7%, odds ratio = 2.392, *P* = 0.078). For patients with first-line SD or PD, no significant difference was observed between the two groups with respect to second-line PFS (median, 4.8 vs. 3.3 months, hazard ratio = 0.862, *P* = 0.521), OS (median, 15.7 vs. 12.4 months, hazard ratio = 0.661, *P* = 0.113), DCR (56.8% vs. 43.9%, odds ratio = 1.681, *P* = 0.234) and ORR (9.1% vs. 4.9%, odds ratio = 1.950, *P* = 0.677) (Supplementary Figure S2).

No other significant predictor was found among these factors (primary tumor site, organs with metastases, number of liver metastatic lesions, first-line chemotherapy regimen, etc.).

### Efficacy of treatment after second-line disease progression

For all patients included in this study, univariate and multivariate Cox regression analyses of OS were conducted after disease progression during second-line treatment. The results showed that second-line continued cetuximab (hazard ratio = 0.663, *P* = 0.034 by Cox regression) and intensive chemotherapy after second-line disease progression (hazard ratio = 0.417, *P* = 0.008 by Cox regression) were independent protective factors for better OS after second-line progression. Bevacizumab administered after second-line progression was a potential protective factor (hazard ratio = 0.644, *P* = 0.084 by Cox regression) (Table 5).

**Table 5:** Univariate and multivariate analyses of overall survival after disease progression during second-line therapy

	Univariate analysis			Multivariate analysis		
	HR	95%CI	*P* value	HR	95%CI	*P* value
Second-line chemotherapy regimen						
FOLFIRI	1	-	-	1	-	-
FOLFOX	0.966	0.677–1.379	0.848	1.082	0.748–1.565	0.675
Second-line continued cetuximab						
No	1	-	-	1	-	-
Yes	0.585	0.407–0.841	0.004*	0.663	0.454–0.969	0.034*
Second-line maintenance treatment						
No	1	-	-	1	-	-
Yes	0.491	0.274–0.881	0.017*	0.745	0.389–1.425	0.373
Following TACE/TAI for metastases						
No	1	-	-	1	-	-
Yes	0.704	0.464–1.067	**0.098**	0.886	0.570–1.376	0.589
Following Radiofrequency for metastases						
No	1	-	-	1	-	-
Yes	1.328	0.746–2.364	0.336	1.278	0.735–2.220	0.385
Following Radiotherapy for metastases						
No	1	-	-	1	-	-
Yes	1.028	0.565–1.870	0.928	1.139	0.615–2.109	0.678
Following chemotherapy						
Best support care	1	-	-	1	-	-
Intensive chemotherapy^§^	0.437	0.233–0.816	0.009*	0.417	0.219–0.792	0.008*
Following bevacizumab						
No	1	-	-	1	-	-
Yes	0.564	0.351–0.906	0.018*	0.644	0.391–1.060	**0.084**

HR: hazard ratio; CI: confidence interval; TACE: transcatheter arterial chemoembolization; TAI: transcatheter arterial infusion.

§ Intensive chemotherapy: receiving at least one intravenous chemotherapy medicine.

*P* values were calculated using Cox regression.

Bold font for *P* < 0.10;

* for *P* < 0.05.

### Safety

For all patients included in this study, information on grade 3 or higher adverse events that occurred during the second-line treatment were collected and analyzed. In general, the observed toxicity was mostly mild in both groups, and no deaths were attributable to second-line treatment. The overall incidence of second-line grade 3 or higher adverse events was 27.5% in cetuximab continuation group and 22.9% in chemotherapy only group (*P* = 0.463). Continued cetuximab significantly increased incidence of newly occurring acne-like rash in patients (10.8% vs. 2.1%, *P* = 0.013). With the exception of the cetuximab-specific acne-like rash, no difference in adverse events was observed between the two groups (22.5% vs. 20.8%, *P* = 0.770). Three (2.9%) patients in the cetuximab continuation group and 2 (2.1%) patients in the chemotherapy only group converted to best supportive care because of they could not tolerate the intensive treatment (Table 6).

**Table 6:** Grade 3 or higher adverse events during second-line therapy

	Cetuximab continuation (*N* = 102)		Chemotherapy only (*N* = 96)		*P* value
	Number	%	Number	%	
Grade 3 or higher adverse event	28	27.5	22	22.9	0.463
Exclude acne-like rash newly occurred	23	22.5	20	20.8	0.770
Details					
Acne-like rash newly occurred	11	10.8	2	2.1	0.013*
Leucopenia/Neutropenia	8	7.8	7	7.3	0.883
Thrombocytopenia	2	2.0	2	2.1	1.000
Diarrhea	4	3.9	4	4.2	1.000
Nausea/Vomiting	6	5.9	5	5.2	0.836
Peripheral neuropathy	7	6.9	5	5.2	0.626
Liver dysfunction	1	1.0	0	0	1.000
Renal dysfunction	0	0	1	1.0	0.485
Intensive treatment intolerance and received best supportive care	3	2.9	2	2.1	1.000

*P* values were calculated using two-sided Pearson's χ^2^ tests or Fisher's exact test for any cell expected count less than 5 or sample size less than 40.

Bold font for *P* < 0.10;

* for *P* < 0.05.

## DISCUSSION

Although there have been considerable advances in the medications used to treat colorectal cancer, the array of anti-cancer agents currently available for clinical use is still very limited. Every kind of medicine that could prolong patients' survival is precious, especially for those with incurable metastases. Second-line treatment with anti-vascular endothelial growth factor (anti-VEGF) antibodies was demonstrated to be effective as a cross-line treatment for mCRC [7, 8]. However, anti-EGFR antibodies were traditionally not considered suitable for continuing use. Several experimental studies reported resistance to anti-EGFR treatment once first-line disease progression had occurred [9–12]. But the clinical evidence was not compelling. Our study addresses this issue by providing important new clinical evidence. We found that for patients with metastatic disease and all RAS wild-type, second-line continued cetuximab significantly improved prognosis, and that these benefits came mainly from patients who had achieved ETS during first-line cetuximab-based treatment.

NRAS and other KRAS (referred to as other RAS) mutations have been shown to be important contraindications for cetuximab [4–6]. In the present study, the results showed that only patients with all RAS wild-type benefited from second-line continued cetuximab. For patients with other RAS mutations, continued cetuximab had no beneficial effect. As a downstream gene of RAS, BRAF was also a promising predictor of the efficacy of cetuximab [13, 14]. In our study (Figure 4), patients with BRAF wild-type could benefit more from cetuximab than those with a BRAF mutation. Unfortunately, the small sample size of BRAF subgroup meant that there was no statistically significant interaction. Further large-scaled studies are needed for confirmation.

Results from earlier studies suggest ETS is an important predictor of prognosis [15, 16]. ETS reflects the treatment efficacy in terms of both speed and depth, and is always more accurate than the traditional ORR. In patients who received first-line cetuximab-based chemotherapy, ETS indicated higher sensitivity to treatment with both targeted agents and cytotoxic chemotherapy. For patients achieving first-line ETS, first-line disease progression was mainly due to resistance to the cytotoxic chemotherapy regimens. Tumor sensitivity to cetuximab was preserved. Indeed, Ekblad et al. [17] observed increased sensitivity to cetuximab in oxaliplatin-resistant cell lines. Thus, the continuation of cetuximab in second-line treatment was more suitable for patients who achieved ETS in first-line cetuximab-based chemotherapy.

For patients who did not achieve ETS during first-line cetuximab-based treatment, second-line continued cetuximab had no beneficial effect. This may reflect acquired resistance to cetuximab. Diaz et al. [10] and Misale et al. [9] reported an induced KRAS mutation rate of 38% to 60% after initial cetuximab-based treatment, which would result in the failure of second-line continued cetuximab. Another explanation could be primary resistance of cetuximab not detected prior to treatment. This could result from other RAS, BRAF, PIK3CA or HER-2 amplification, and from other gene mutations [13, 14, 18, 19] or intratumoral heterogeneity [20]. For this reason, more detailed gene analysis is needed before applying cetuximab.

As a retrospective study, there were potential imbalances between the two test groups. To reveal and reduce this interference, all patients included in this study received first-line and second-line treatment strictly according to the protocols developed by the MDT. A precise follow-up was done to avoid missing any data related to the treatment. All potential factors were listed and compared in a baseline analysis. Multivariate analysis was also conducted for second-line PFS and OS after second-line disease progression. These analyses proved that during first-line and second-line treatment, there was no significant imbalance between the two test groups. However, there were potential imbalances in the treatment after second-line disease progression. The efficacy of second-line continued cetuximab was reliable in both PFS and OS, and was not significantly interfered with by these imbalances.

In summary, despite several drawbacks, our study showed that for patients with all RAS wildtype and initially unresectable mCRC who experienced disease progression during standard first-line cetuximab-based treatment, continuation of cetuximab was effective and safe as second-line treatment. ETS in first-line cetuximab-based treatment could be a significant predictor of the efficacy of second-line continued cetuximab. Therefore, the indications for cetuximab might be prudently expanded, and a further large randomized clinical trial should be conducted to confirm these conclusions.

## MATERIALS AND METHODS

### Study eligibility

Consecutive patients presenting between January 2012 and January 2015 with unresectable mCRC were retrospectively identified from the colorectal cancer database in Zhongshan Hospital, Fudan University (Shanghai, China). For these patients, disease evaluations and treatment strategies were conducted at multidisciplinary team (MDT) meetings.

The enrollment was set at the beginning of second-line treatment. The inclusion criteria were as follows: aged from 18 to 75 years; Eastern Cooperative Oncology Group (ECOG) performance status of 0 to 2; pathologically confirmed primary colorectal cancer; unresectable mCRC based on clear radiographic evidence as determined by an MDT; wild-type KRAS exon 2 codons 12 and 13; received cetuximab plus mFOLFOX6 (fluorouracil, leucovorin, and oxaliplatin) or FOLFIRI (fluorouracil, leucovorin, and irinotecan) as first-line treatment for at least 4 cycles; first-line disease progression assessed by an MDT; received second-line treatment within 4 weeks after progression on first-line treatment; received a different chemotherapy regimen (first-line mFOLFOX6 converted to second-line FOLFIRI; first-line FOLFIRI converted to second-line mFOLFOX6) alone or with continued cetuximab as second-line treatment for at least 4 cycles. Patients were excluded if they exhibited tumor peritoneal dissemination, first-line treatment intolerance or other cancers within previous 5 years of the end of follow-up (with the exception of squamous cell carcinoma of skin and cervical cancer *in situ*). Patients were also excluded if they were unable to afford cetuximab as second-line treatment. The chemotherapy regimen CapeOX (capecitabine and oxaliplatin), bevacizumab and other targeted agents, transcatheter arterial chemoembolization (TACE), transcatheter arterial infusion (TAI), radiotherapy and radiofrequency ablation were not permitted until second-line disease progression. Eligible patients were divided into two groups according to their second-line treatment: patients receiving continued cetuximab plus changed chemotherapy were the cetuximab continuation group, and patients receiving only changed chemotherapy were the chemotherapy only group.

The resectability of liver metastases was determined based on the ability to obtain a complete resection (i.e., negative margins), preserve an adequate liver remnant (> 30% of healthy liver), and preserve adequate vascular inflow and outflow as well as biliary drainage [3]. The resectability of lung metastases was determined based on the ability to obtain complete resection (i.e., negative margins) based on the anatomic location and the extent of disease and ability to maintain adequate lung function [21, 22]. Metastases were deemed unresectable if the above criteria were not met. Bone/brain metastases and peritoneal disease were defined as unresectable.

This study was approved by the institutional review board of Zhongshan Hospital, Fudan University. The investigators obtained informed consent from each patient.

### RAS and BRAF mutation analysis

KRAS exon 2 (codon 12/13) and BRAF mutations were analyzed before the first-line treatment. Colonoscopic biopsy of the primary tumors was conducted. DNA was extracted from formalin-fixed paraffin-embedded (FFPE) tumor tissues obtained at biopsy. The mutation status of KRAS exon 2 (codon 12 and 13) was assessed using polymerase chain reaction clamping and pyrosequencing techniques. BRAF mutation (V600E) was assessed using a similar approach.

Other RAS gene detection was conducted as a post hoc analysis during or after the follow-up was over. Samples were FFPE specimens from colonoscopic biopsy or primary tumor resections. Mutation status of KRAS exons 3 (codon 59 and 61) and exon 4 (codon 117 and 146), NRAS exons 2 (codon 12 and 13), exon 3 (codon 59 and 61) and 4 (codon 117 and 146) were also assessed using approaches similar to those mentioned above. All gene detection protocols were performed by Gene Tech (Shanghai) Company Limited.

### Chemotherapy regimen

In this study, chemotherapy (mFOLFOX6, FOLFIRI) and cetuximab were given as previously reported [3]. Intensive combination therapy was sustained until disease progression or adverse events were intolerable. In patients whose tumors remained stable and who experienced no tumor-associated symptoms for more than 4 months (8 cycles), maintenance treatment became a possible alternative to intensive combination therapy. During the maintenance treatment, fluorouracil/leucovorin was given instead of mFOLFOX6 or FOLFIRI. In patients with adverse events were ≥ Grade 3, the intensive combination therapy was suspended, and symptomatic treatment was given. Maintenance treatment was also used in patients who were still unable to tolerate the intensive treatment after a rest. If the disease progressed during the maintenance treatment, the previous intensive treatment was attempted again.

### Outcome assessments

Tumor status (complete response, CR; partial response, PR; stable disease, SD; progressive disease, PD) was evaluated according to the Response Evaluation Criteria in Solid Tumors (RECIST) V 1.1 [23]. All endpoints in this study were assessed from the start of the second-line treatment, including PFS, OS, overall response rate (ORR, CR + PR), disease control rate (DCR, CR + PR + SD), and safety end points. Early tumor shrinkage (ETS) at 8 weeks was defined as a relative shrinkage of ≥ 20% of the sum of the longest diameters of the target lesions compared to the baseline. Tumor progression during maintenance treatment was not considered PD until the previous intensive combination therapy also failed.

Colonoscopic biopsies were used to diagnose the primary tumor and recurrence. Enhanced CT/MRI/PET-CT scans were used to identify the metastases and peritoneal dissemination. The pathological tumor stage was documented according to the AJCC TNM classification (version 7, 2010). Once the cases were discussed at the MDT meeting, strict follow-up was conducted: the carcino-embryonic antigen (CEA) and abdominal ultrasound were performed every 4 weeks, and enhanced CT/MRI was performed every 8 weeks. Follow-up evaluations by the MDT were conducted every 8 weeks, or at any time, if necessary. The end of follow-up was set at January 2015. Adverse events were categorized according to the National Cancer Institute Common Toxicity Criteria, version 3.0.

### Statistical analysis

Patient baseline characteristics and disease factors were summarized using descriptive statistics. The categorical parameters were compared using two-sided Pearson's χ^2^ test or Fisher's exact test as appropriate, with odds ratios and *P* value. All summary statistics on time-to-event variables were calculated according to the Kaplan-Meier method. Hazard ratios were determined using Cox regression, and *P* values using the log-rank test. Patients lost to follow-up were addressed as censored data in survival analysis. In subgroup analysis, Cox regression was used for interaction analysis. Cox regression was also used in univariate and multivariate analysis. The follow-up time was reported with an interquartile range (IQR). SPSS software (V 16.0; SPSS, Chicago, IL, USA) was used for all statistical analyses. All *P* values were two-sided and were considered significant when < 0.05.

## SUPPLEMENTARY MATERIALS FIGURES AND TABLE


