# Supplementary material for: Efficacy of continued cetuximab for unresectable metastatic colorectal cancer after disease progression during first-line cetuximab-based chemotherapy: a retrospective cohort study
Source: Oncotarget. 2016 Feb 4;7(10):11380–96. doi: 10.18632/oncotarget.7193 (PMC4905480; doi:10.18632/oncotarget.7193)
Supplement: Supplementary file 1 [file oncotarget-07-11380-s001.pdf]

Efficacy of continued cetuximab for unresectable metastatic colorectal cancer after disease progression during first-line cetuximab-based chemotherapy: a retrospective cohort study

Supplementary Materials

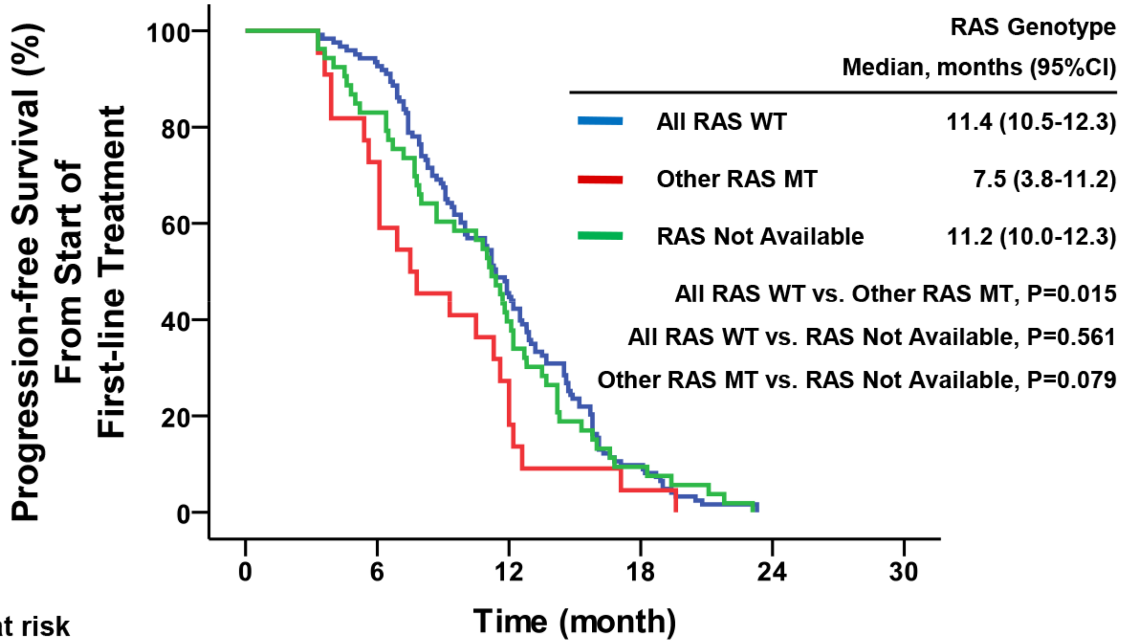

No. at risk						
All RAS WT	123	115	56	12	0	0
Other RAS MT	22	16	6	1	0	0
RAS Not Available	53	44	21	5	0	0

Supplementary Figure S1: Patients with other RAS mutation had poorer first-line PFS. Kapian-Meier curves of first-line progression- free survival with standard first-line cetuximab-based treatment. Patients with all RAS wild type had significantly better first-line progression-free survival than patients with other RAS mutation. WT: wild type; MT: mutant type. P: log-rank test.

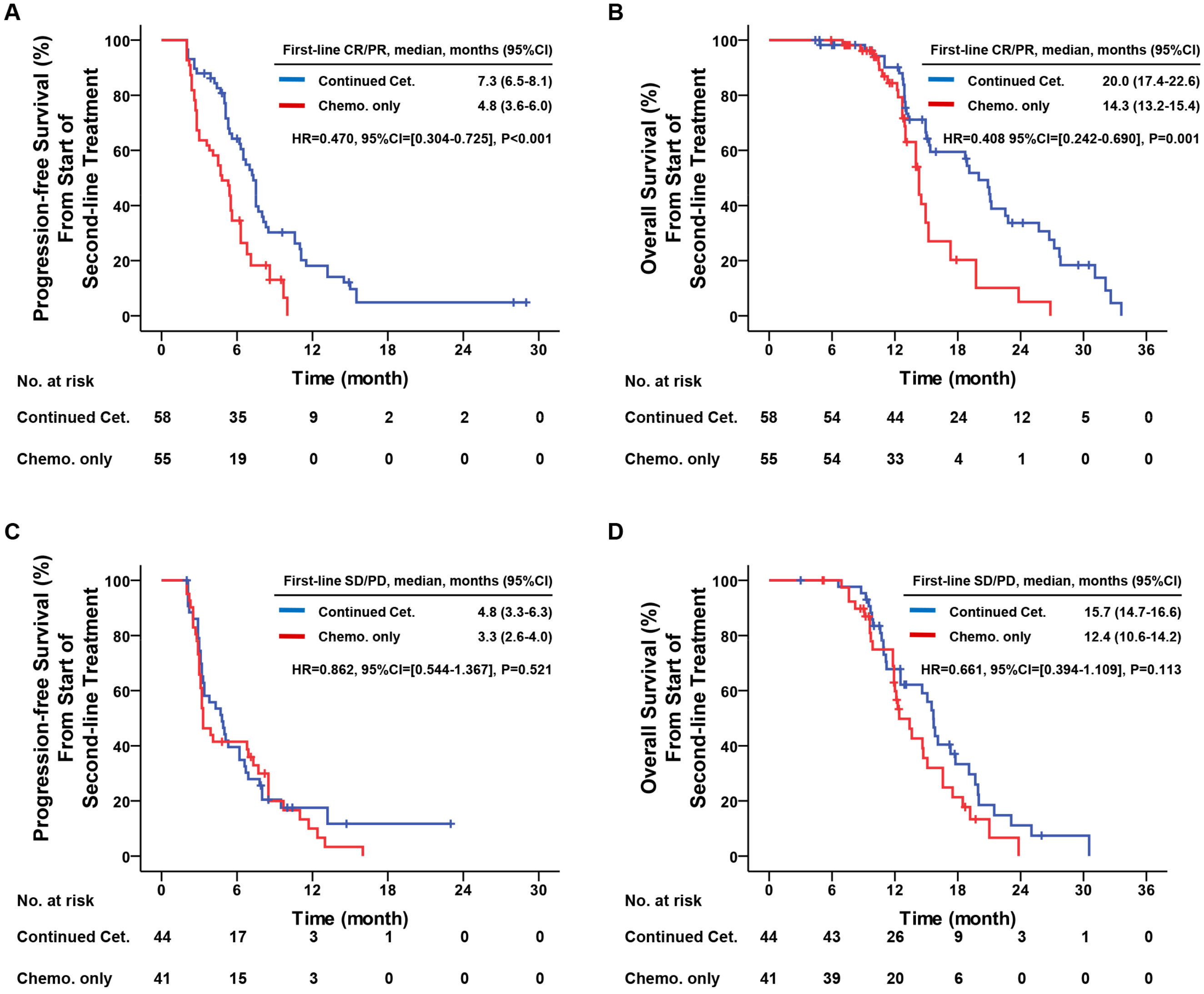

Supplementary Figure S2: First-line overall response as a predictor for the efficacy of second-line treatment. (**A**) Kaplan-Meier progression-free survival and (**B**) overall survival curves for patients reached CR/PR in first-line treatment, (**C**) progression-free survival and (**D**) overall survival curves for patients with first-line SD/PD, comparing the continued cetuximab plus changed chemotherapy and changed chemotherapy only as second-line treatment. Cet.: Cetuximab; Chemo.: chemotherapy; CR: complete response; PR: partial response; SD: stable disease; PD: progressive disease; HR hazard ratio; Cl: confidence interval. *P* value: by log-rank test.

Supplementary Table S1: Univariate and multivariate analysis for second-line progression-free survival

	Second-line median PFS (month)	Univariate analysis			Multivariate analysis		
		**HR**	**95% CI**	***P* value**	**HR**	**95% CI**	***P* value**
Age - year							
≤ 55	5.4	1	-	-	1	-	-
> 55	5.3	1.183	0.872–1.605	0.280	1.269	0.915–1.761	0.154
Sex							
Male	5.6	1	-	-	1	-	-
Female	5.0	1.274	0.927–1.750	0.135	1.331	0.932–1.901	0.116
ECOG performance status							
0	5.5	1	-	-	1	-	-
1–2	5.3	1.123	0.826–1.528	0.459	1.098	0.421–1.056	0.586
Primary tumor site							
Right-sided colon	4.8	1	-	-	1	-	-
Left-sided colon	7.1	0.660	0.442–0.987	**0.043***	0.667	0.421–1.056	**0.084**
Rectum	5.3	1.016	0.714–1.446	0.928	1.051	0.702–1.574	0.808
Organs with metastases							
Liver only	5.5	1	-	-	1	-	-
Liver plus others	5.3	1.030	0.711–1.494	0.874	0.925	0.622–1.376	0.701
Non-liver	5.1	0.973	0.493–1.921	0.937	0.817	0.399–1.674	0.581
Other RAS genotype							
Wild type	6.3	1	-	-	1	-	-
Mutant type	3.3	1.807	1.124–2.906	**0.015***	2.141	1.285–3.569	**0.003***
Not available	3.9	1.178	0.832–1.669	0.355	1.221	0.837–1.782	0.299
BRAF genotype							
Wild type	5.6	1	-	-	1	-	-
Mutant type	3.2	2.859	1.615–5.063	**< 0.001***	3.001	1.639–5.494	**< 0.001***
Primary tumor resection							
No	4.7	1	-	-	1	-	-
Yes	5.5	0.964	0.685–1.357	0.834	0.813	0.566–1.170	0.265

In first-line cetuximab-based treatment							
Chemotherapy regimen							
mFOLFOX6	5.6	1	-	-	1	-	-
FOLFIRI	4.8	1.058	0.778–1.439	0.721	0.903	0.635–1.284	0.570
Maintenance treatment							
No	5.3	1	-	-	1	-	-
Yes	5.5	1.015	0.732–1.407	0.929	1.086	0.693–1.702	0.719
Early tumor shrinkage							
No	4.8	1	-	-	1	-	-
Yes	6.3	0.709	0.522–0.963	**0.028***	0.682	0.473–0.984	**0.041***
Overall response							
SD or PD	4.1	1	-	-	1	-	-
CR or PR	5.6	0.852	0.629–1.155	0.303	0.809	0.504–1.298	0.379
Acne-like rash, grade							
0–1	5.3	1	-	-	1	-	-
≥ 2	5.5	1.002	0.733–1.370	0.991	1.125	0.782–1.617	0.527
Second-line continued cetuximab							
No	4.5	1	-	-	1	-	-
Yes	6.3	0.646	0.476–0.877	**0.005***	0.675	0.491–0.930	**0.016***

HR: hazard ratio; CI: confidence interval.
ECOG: Eastern Cooperative Oncology Group.
Other RAS including KRAS exons 3 (codon 59 and 61) and exon 4 (codon 117 and 146), NRAS exons 2 (codon 12 and 13), exon 3 (codon 59 and 61) and 4 (codon 117 and 146).
P values were calculated using Cox regression.
Bold font for $P < 0.10$; * for $P < 0.05$.
